# Association Between Post‐Partum Anaemia and Depressive Symptoms at Two Months After Vaginal Delivery: A Secondary Analysis of the TRAAP Trial

**DOI:** 10.1111/1471-0528.18289

**Published:** 2025-07-14

**Authors:** Lola Loussert, Loïc Sentilhes, Alizée Froeliger, Aurélien Seco, Marie Pierre Bonnet, Sarah Tebeka, Catherine Deneux‐Tharaux, the TRAAP study group, Loïc Sentilhes, Norbert Winer, Elie Azria, Marie Victoire Sénat, Camille Le Ray, Delphine Vardon, Franck Perrotin, Raoul Desbrière, Florent Fuchs, Gilles Kayem, Guillaume Ducarme, Muriel Doret‐Dion, Cyril Huissoud, Caroline Bohec, Philippe Deruelle, Astrid Darsonval, Jean‐Marie Chrétien, Aurélien Seco, Valérie Daniel

**Affiliations:** ^1^ Université Paris Cité Institut Santé Des Femmes, Centre for Research in Epidemiology and Statistics (CRESS) U1153, Obstetrical Perinatal and Pediatric Epidemiology Research Team (Epopé), INSERM, INRAE Paris France; ^2^ Department of Obstetrics and Gynecology Toulouse University Hospital Toulouse France; ^3^ Department of Obstetrics and Gynecology Bordeaux University Hospital Bordeaux France; ^4^ Clinical Research Unit Necker Cochin APHP Paris France; ^5^ Department of Anesthesiology and Intensive Care Medicine, Armand Trousseau Hospital, DMU DREAM, GRC 29, Assistance Publique‐Hôpitaux de Paris Sorbonne University Paris France; ^6^ Department of Psychiatry Louis Mourier Hospital Colombes France; ^7^ Université Paris Cité, INSERM UMR1266, Institute of Psychiatry and Neuroscience of Paris Paris France

**Keywords:** maternal mental health, post‐partum anaemia, post‐partum depression, post‐partum haemoglobin level, vaginal delivery

## Abstract

**Objective:**

To assess the association between maternal haemoglobinaemia in the immediate post‐partum period and PPD symptoms 2 months after vaginal delivery.

**Design:**

Ancillary cohort study of the TRAAP trial, a multicentre trial.

**Setting:**

In France, 2015–2016.

**Population:**

Women with a singleton pregnancy and vaginal delivery ≥ 35 weeks. We excluded women with known psychiatric conditions and those who were non‐respondent to the Edinburgh Post‐partum Depression Scale (EPDS) questionnaire.

**Methods:**

The exposure was immediate post‐partum haemoglobinemia (systematically collected in TRAAP trial) as a continuous variable.

**Main Outcome:**

PPD symptoms at 2 months post‐partum, defined as an EPDS score ≥ 11. We also differentiated two levels of PPD symptom severity: moderate (11 ≤ EPDS < 13) and severe (EPDS ≥ 13) depressive symptoms.

**Results:**

Amongst the 2672 women included, 1115 (43.6%) had post‐partum anaemia (haemoglobin < 11 g/dL) in the immediate post‐partum and 369 (13.8%) had PPD symptoms at 2 months. The relation between haemoglobin and PPD symptoms was linear. In the multivariable analysis, each 1 g/dL increase in haemoglobin level was associated with a 9% decrease in the risk of post‐partum depression symptoms (adjusted Relative Risk 0.91; 95% CI 0.82–0.997). Post‐partum haemoglobin was specifically associated with moderate depressive symptoms (adjusted Relative Risk 0.84; 95% CI, 0.72–0.98) but not with severe depressive symptoms (aRR 0.95; 95% CI, 0.84–1.07).

**Conclusions:**

In women with vaginal delivery, each 1 g/dL increase in haemoglobin level was associated with a 9% decrease in the risk of post‐partum depression symptoms.

## 1 Introduction

Post‐partum depression (PPD) affects 10% to 25% of women worldwide [1, 2], and is associated with adverse outcomes for both the women and the child [1, 3]. PPD is the main underlying condition of maternal suicide, one of the leading causes of maternal mortality in high‐income countries, such as the United Kingdom and France [4, 5]. PPD alters mother–child interactions and is associated with increased rates of impaired neurobehavioural development and psychiatric disorders in the child [6, 7, 8, 9]. Prevention of PPD requires an understanding of its risk factors. The main reported risk factors for PPD are related to psychosocial vulnerability [9]. The impact of the obstetrical context remains less documented, although potentially more actionable.

Anaemia affects about 500 million women of reproductive age worldwide [10]. It is particularly common in the post‐partum period, affecting up to 50% of women in high‐income countries [11, 12] and 50% to 80% and treatment, iron supplementation.

Anaemia can cause fatigue [13], decreased physical performance, and impaired cognition [14], which may promote PPD. Moreover, iron is involved in the synthesis of neurotransmitters such as dopamine, which is implicated in depression pathophysiology [15]. In the existing literature, the association between maternal anaemia and PPD is inconsistent [16]. The heterogeneous assessment of anaemia and of PPD, in terms of timing, diagnosis tool, and cut‐off values, may account for these discrepancies [17]. Moreover, potential confounding factors such as maternal chronic conditions, pregnancy, and delivery characteristics are not always taken into account [16]. A recent umbrella meta‐analysis of observational studies concluded that anaemia seems to increase the risk of PPD, with a low to very low level of evidence due to the poor quality of data [9]. Additionally, most prior studies were conducted in middle‐ and low‐income countries, limiting their applicability to high‐income populations, where anaemia prevalence differs widely. If the role of anaemia in PPD were to be confirmed by high‐quality evidence, maternal anaemia could then be a firm modifiable risk factor for PPD of great interest.

We hypothesised that post‐partum haemoglobin in the immediate post‐partum period is associated with PPD symptoms at 2 months. The TRanexamic Acid for Preventing Post‐partum Haemorrhage After Vaginal Delivery (TRAAP) trial [18] which tested tranexamic acid to prevent post‐partum haemorrhage amongst women who had a vaginal delivery, systematically collected haemoglobin in the immediate post‐partum period and assessed post‐partum mental health status two months after delivery. Therefore, it offered the opportunity to test our hypothesis.

Our objective was to assess the association between haemoglobin in the immediate post‐partum period and post‐partum depression symptoms at two months in women with vaginal delivery.

## 2 Materials and Methods

### 2.1 Study Design and Population

This study was an ancillary planned analysis of the TRAAP trial, conducted in 2015–2016 in 15 French maternity units [18]. This double‐blinded randomised controlled trial (RCT) assessed the impact of tranexamic acid for the prevention of PPH after vaginal delivery. Women were randomised to receive 1 g of tranexamic acid or placebo after delivery. This RCT found no significant difference in the primary outcome: PPH defined as a blood loss of at least 500 mL. It enrolled women in labour aged 18 or more who had a planned vaginal birth of a singleton live foetus at 35 or more weeks of gestation. Exclusion criteria of TRAAP were detailed in previous articles [19].

This secondary analysis included all women in the modified intention to treat population (randomised women who underwent vaginal delivery) of the TRAAP trial without a known psychiatric condition and who responded to the self‐administered questionnaire sent two months after delivery.

### 2.2 Data Collection

After randomisation, the midwife or obstetrician handling the delivery prospectively collected the characteristics of labour, delivery, and third stage of labour, including blood loss (the main outcome of the TRAAP trial), quantified with a collector bag. Post‐partum characteristics were also prospectively collected, including neonatal status. The women's other characteristics, particularly their psychiatric history, were collected from a manual review of the medical charts by a research assistant independent of the local medical team. The quality of collected data was checked in each centre for a 10% random selection of the women included and for all women with PPH. Two months after childbirth, all women received by electronic or postal mail a self‐administered questionnaire in French, including the Edinburgh Post‐partum Depression Scale (EPDS). At 3 months post‐partum, women were contacted for a telephone interview to assess the trial drug's potential adverse events as part of the TRAAP trial protocol. On this occasion, women who had not responded to the psychological questionnaires were asked to complete and return them.

### 2.3 Exposure

The exposure of interest was post‐partum haemoglobin level, assessed in the 5 days following delivery. The trial's protocol included assessment of maternal haemoglobin at Day 2 post‐partum. If missing, it was measured on day 3 [19]. In cases where haemoglobin was assessed several times in the 5 days following delivery, we used the last haemoglobinemia as it best reflects subsequent maternal exposure over the 2 months post‐partum. All women who received a red blood cells transfusion in the immediate post‐partum had a post‐transfusion haemoglobin assessment which was the one considered in the analysis.

### 2.4 Outcome

The primary outcome was symptoms of post‐partum depression defined as an EPDS (Edinburgh Postnatal Depression Scale) score of eleven or more, 2 months after delivery. The EPDS is the most commonly used PPD screening tool and is recommended in the American College of Obstetrics and Gynaecology guidelines published in 2023 [20]. It is a 10‐item self‐administered questionnaire, with a score from 0 to 30, validated in French language [21]. The EPDS is a screening tool and does not provide a definite diagnosis; that is why we used the terms ‘PPD symptoms’ A recent meta‐analysis concluded that combined sensitivity and specificity for the EPDS is maximised at a cut‐off value of 11 or higher (sensitivity 81% and specificity 88% for semi structured interviews) [22]. As Levis et al. proposed, we also differentiated two levels of PPD symptoms severity: moderate (11 ≤ EPDS < 13) and severe (EPDS ≥ 13), the specificity of the 13 cut‐off being higher [22].

### 2.5 Statistical Analyses

First, we compared the characteristics of the women who responded to the EPDS questionnaire with those of the women who did not. We described the timing of post‐partum haemoglobin assessment and the distribution of post‐partum haemoglobin in the study population. Then, we compared the characteristics of the women, pregnancies, and deliveries amongst women with and without post‐partum anaemia, using the Chi squared test and *t* test. Post‐partum anaemia was defined as immediate post‐partum haemoglobin of less than 11 g/dL, in accordance with French guidelines [23] and with WHO definition of gestational anaemia [24]. Haemoglobin thresholds used to define post‐partum anaemia vary worldwide from 10 (UK guidelines [25]) to 12 g/dL (USA CDC recommendation [26]). However, since our main exposure was haemoglobin level as a continuous variable, the threshold used to define anaemia did not impact our association analysis.

To assess the association between post‐partum haemoglobin and symptoms of PPD, we used univariable then multivariable robust Poisson regression. The relation between post‐partum haemoglobin and PPD symptoms was assessed with fractional polynomial modelling and was found to be log linear (Figure S1). Post‐partum haemoglobin was then treated as a continuous variable without transformation. A directed acyclic graph (Figure S2), based on literature review and authors expertise, was used to represent causal assumptions between post‐partum haemoglobin, PPD symptoms, and covariates and identify potential confounding factors. Maternal confounders included in the multivariable analysis were age, body mass index, parity, place of birth, chronic condition, gestational diabetes, and preeclampsia. Other confounders included in the analysis were: induction of labour, duration of labour, instrumental delivery, and episiotomy. The relations between PPD symptoms and the 3 continuous covariables (maternal age, maternal body mass index and duration of labour) were not linear, so these three variables were transformed into second‐degree fractional polynomials. We tested for effect modification, with likelihood ratio test, for two clinically relevant variables: parity and maternal place of birth. We found positive effect modification of haemoglobin level across strata of maternal place of birth on an additive scale (Relative Excess Risk due to Interaction RERI (95% CI) 0.1250 (0.0321–0.2179)). Therefore, analyses were rerun after stratification for maternal place of birth (European‐born women and non‐European‐born women). There were 2195 (82.1%) women with no missing data for covariates included in the multivariable model. The proportion of women with missing data for any covariate ranged from 0% to 9.6%. Characteristics of the women with complete data were similar to those of women with missing data (Table S1). We used multiple imputation chained equations to impute missing data in 20 independent data sets.

In a first sensitivity analysis, we used inverse probability weighting (IPW) to address attrition bias due to non‐respondents to the EPDS questionnaire. We hypothesised that non‐response to the EPDS at 2 months post‐partum depended on observed pre‐existing characteristics of the women, characteristics related to pregnancy, labour, delivery and immediate post‐partum. This method weighted respondents by the inverse of their probability of responding to the EPDS at 2 months. Consequently, a woman with a higher probability of responding would have a lower weight in the analysis.

We performed a second sensitivity analysis after exclusion of the women with post‐partum haemorrhage, defined by blood loss of 500 mL or more according to French guidelines [27]. Indeed, on the one hand, PPH decreases post‐partum haemoglobin. On the other hand, medical interventions performed to treat PPH could affect women's experience of childbirth and may promote PPD [28]. Therefore, we wanted to verify that the association remained unchanged in women who did not experience post‐partum haemorrhage.

In the third sensitivity analysis, the outcome was EPDS score as a continuous variable. Because the relation between post‐partum haemoglobin and EPDS score was log‐linear, EPDS score was treated as a continuous variable without transformation.

All analyses were performed with STATA 16. Graphics were created with R software version 4.0.4, package ‘ggplot2’.

## 3 Approval and Funding

TRAAP trial was approved by the West II Committee for the Protection of Research Subjects (Ethics Committee) and by the French Health Products Safety Agency (2014‐001748‐39). TRAAP trial was supported by a grant from the French Ministry of Health (PHRCN 1370458N). This secondary analysis received no funding.

## 4 Results

**FIGURE 1:**
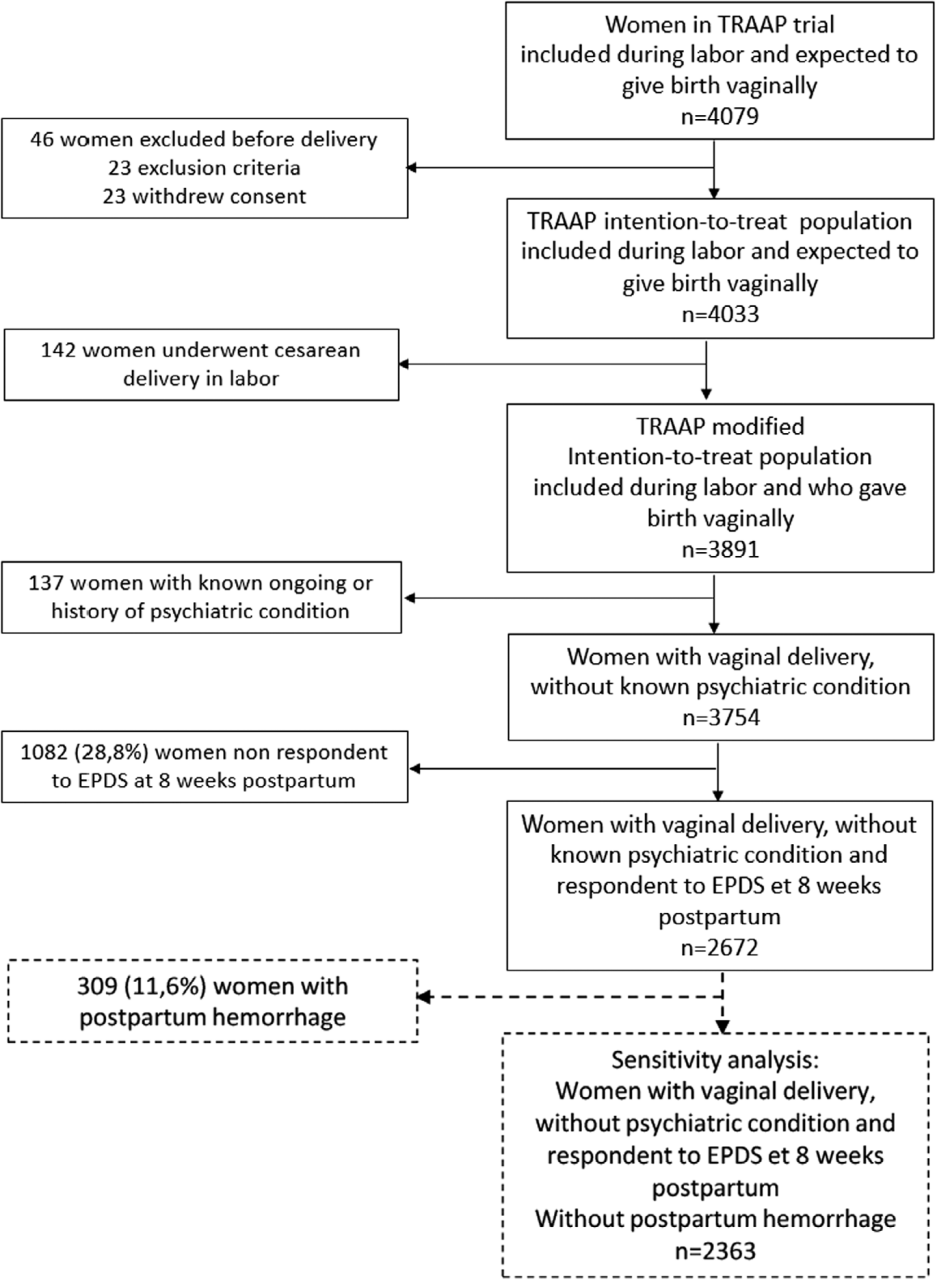
Study population. EPDS, Edinburgh Postpartum Depression Scale.

Amongst the 3891 women of the TRAAP trial who gave birth vaginally, we excluded 137 women with known psychiatric conditions and 1082 (28.8%) women who did not complete the EPDS. The analysis then included 2672 women (Figure 1).

Respondents to EPDS, compared to non‐respondents, were more often born in Europe, more often primiparous and with a history of abortion. Post‐partum anaemia was more frequent in non‐respondents to EPDS than in respondents (Table S2).

In the study population, median post‐partum haemoglobin, assessed within the 5 days following delivery, was 11.2 g/dL and 1115 (43.6%) women had post‐partum anaemia (Table 1).

**TABLE 1:** Haemoglobin in the immediate post‐partum in the study population (*n* = 2672).

Haemoglobin in the immediate post‐partum^a^	*n* (%)
Timing of post‐partum haemoglobin assessment	
Day 1	136 (5.3)
Day 2	2248 (87.8)
Day 3	103 (4.0)
Day 4	59 (2.3)
Day 5	14 (0.5)
Post‐partum haemoglobin (g/dL) median (IQR)	11.2 (10.2–12.0)
Post‐partum anaemia (Hb < 11 g/dL)	1115 (43.6)
Post‐partum haemoglobin (g/dL)	
≥ 11	1445 (56.4)
[10–11[	621 (24.3)
[9–10[	329 (12.9)
[8–9	133 (5.2)
< 8	32 (1.3)

Abbreviations: dL, deciliter; g, grams; IQR, interquartile range.

^a^ Defined as the latest maternal haemoglobin measured in the 5 days following delivery. In case of red blood cell transfusion, haemoglobin after transfusion was considered. One hundred and twelve women had missing data on post‐partum haemoglobin.

Women with post‐partum anaemia, compared to women without, were more often primiparous and more often hospitalised during pregnancy. They more often had an induction of labour, a longer active phase of labour, an instrumental delivery, an episiotomy, a newborn weighing 4000 g or higher, and a post‐partum haemorrhage (Table 2).

**TABLE 2:** Maternal, pregnancy, labour and delivery characteristics in women of the study population, overall and according to post‐partum anaemia.

	Overall population *n* = 2672, *n* (%)	Women with post‐partum anaemia^a^ *n* = 1115, *n* (%)	Women without post‐partum anaemia^a^ *n* = 1445, *n* (%)
**Maternal characteristics**			
Age (*n* = 2672) (years)			
< 25	216 (8.1)	108 (9.7)	107 (7.4)
25–29	877 (32.8)	375 (33.6)	475 (32.9)
30–34	1073 (40.2)	423 (37.9)	594 (41.1)
≥ 35	506 (18.9)	209 (18.7)	269 (18.6)
BMI (*n* = 2654) (kg.m^−2^)			
< 18.5	175 (6.6)	69 (6.2)	99 (6.9)
[18.5–25]	1801 (67.9)	738 (66.7)	986 (68.7)
]25–30]	459 (17.3)	204 (18.4)	238 (16.6)
]30–35]	160 (6.0)	77 (7.0)	76 (5.3)
> 35	59 (2.2)	19 (1.7)	36 (2.5)
Place of birth (*n* = 2561)			
Europe	2287 (89.3)	934 (87.5)	1256 (90.8)
Sub Saharan Africa	68 (2.7)	30 (2.8)	34 (2.5)
North Africa	142 (5.5)	70 (6.6)	65 (4.7)
Asia	44 (1.7)	23 (2.2)	20 (1.5)
Other	20 (0.8)	10 (0.9)	8 (0.6)
Pre conception smoking (*n* = 2665)	644 (24.2)	253 (22.8)	360 (25.0)
**Pre‐existing conditions** (*n* = 2672)			
Any pre‐existing condition	27 (1.0)	13 (1.2)	12 (0.8)
Chronic hypertension	13 (0.5)	8 (0.7)	3 (0.2)
Diabetes	12 (0.5)	3 (0.3)	9 (0.6)
**Obstetrical history**			
Previous abortion (*n* = 2672)	336 (12.6)	150 (13.5)	170 (11.8)
Parity (*n* = 2672)			
Primiparous	1485 (55.6)	691 (62.0)	727 (50.3)
Multiparous without previous caesarean	1037 (38.8)	341 (30.6)	657 (45.4)
Multiparous with previous caesarean	150 (5.6)	83 (7.4)	61 (4.2)
History of PPH (*n* = 2672)	128 (4.8)	58 (5.2)	65 (4.5)
**Pregnancy complications**			
Thrombopenia (< 150 000/mm^3^) (*n* = 2672)	118 (4.4)	45 (4.0)	71 (4.9)
Gestational diabetes (*n* = 2672)	280 (10.5)	104 (9.3)	162 (11.2)
Preeclampsia (*n* = 2672)	17 (0.6)	10 (0.9)	7 (0.5)
**Labour and delivery characteristics**			
Induction of labour (*n* = 2672)	522 (19.5)	251 (22.5)	255 (17.7)
Oxytocin during labour (*n* = 2672)	1582 (59.2)	754 (67.6)	769 (53.2)
Duration of active labour (h)^b^ (*n* = 2413)	4.3 (2.6)	4.9 (2.8)	3.9 (2.3)
Instrumental vaginal delivery (*n* = 2672)	485 (18.2)	281 (25.2)	188 (13.0)
Episiotomy (*n* = 2672)	668 (25.0)	398 (35.7)	242 (16.8)
Post‐partum blood loss (mL)^c^ (*n* = 2658)			
< 500	2363 (88.9)	869 (78.3)	1388 (96.5)
[500–1000]	195 (7.3)	152 (13.7)	40 (2.8)
> = 1000	100 (3.8)	89 (8.0)	10 (0.7)
Birthweight ≥ 4000 g (*n* = 2672)	203 (7.6)	114 (10.2)	82 (5.7)

Abbreviations: BMI, body mass index; dL, deciliters; g, grams; h, hours.

^a^ Post‐partum anaemia: haemoglobin < 11 g/dL^−1^ in the immediate post‐partum. Among the 2672 women included in the analysis, 112 women had missing data on post‐partum haemoglobin.

^b^ mean (SD).

^c^ Quantified with a graduated collector bag (mL).

The prevalence of post‐partum depression symptoms at 2 months post‐partum according to haemoglobin in the immediate post‐partum is shown in Figure S3.

At 2 months post‐partum, 369 (13.8%) women had symptoms of PPD. After adjustment for confounders, immediate post‐partum haemoglobin was associated with symptoms of PPD: each 1 g/dL increase in haemoglobin level was associated with a 9% decrease in the risk of post‐partum depression symptoms (adjusted RR 0.91; 95% CI, 0.82–0.997) (Table 3).

**TABLE 3:** Association between haemoglobin in the immediate post‐partum and depression symptoms at 2 months post‐partum, overall and according to maternal place of birth.

	Prevalence of the outcome *n* (%)	RR^a^ For each 1 g/dL increase in Hb	aRR^b^ For each 1 g/dL increase in Hb	aRR^c^ For each 1 g/dL increase in Hb
**Overall population** *n* = 2672				
**PPD symptoms (EPDS ≥ 11)**	369 (13.8)	0.92 [0.84–0.995]	0.89 [0.80–0.987]	0.91 [0.82–0.997]
**PPD symptoms**				
None^d^	2303 (86.2)	Ref	Ref	Ref
Moderate symptoms^e^	139 (5.2)	0.90 [0.79–1.02]	0.81 [0.69–0.95]	0.84 [0.72–0.97]
Severe symptoms^f^	230 (8.6)	0.93 [0.84–1.03]	0.94 [0.83–1.08]	0.95 [0.84–1.07]
**According to maternal place of birth** ^g^				
**European‐born women** *n* = 2287				
**PPD symptoms (EPDS ≥ 11)**	282 (12.3)	0.92 [0.84–1.01]	0.89 [0.80–0.99]	0.91 [0.83–1.01]
**PPD symptoms**				
None^d^	2005 (87.7)	Ref	Ref	Ref
Moderate symptoms^e^	112 (4.9)	0.87 [0.76–1.01]	0.79 [0.67–0.93]	0.84 [0.72–0.98]
Severe symptoms^f^	170 (7.4)	0.95 [0.85–1.07]	0.96 [0.84–1.10]	0.97 [0.85–1.10]
**Non‐European‐born women** *n* = 274				
**PPD symptoms (EPDS ≥ 11)**	63 (23.0)	1.04 [0.84–1.28]	0.98 [0.76–1.26]	0.97 [0.77–1.24]
**PPD symptoms**				
None^d^	211 (77.0)	Ref	Ref	Ref
Moderate symptoms^e^	20 (7.3)	1.26 [0.89–1.77]	1.27 [0.85–1.90]	1.29 [0.88–1.91]
Severe symptoms^f^	43 (15.7)	0.94 [0.73–1.21]	0.83 [0.61–1.13]	0.83 [0.62–1.12]

*Note:* *Overall population n* = 2195 for complete case analysis and *n* = 2672 after multiple imputation; multivariable robust Poisson regression models including maternal age, BMI, parity, place of birth, chronic condition, gestational diabetes, preeclampsia, induction of labour, duration of labour, instrumental delivery, episiotomy. **Analyses according to maternal place of birth:** *European‐born women n* = 1950 (complete case analysis) and 2287 (after multiple imputation) *Non‐European‐born women* and *n* = 243 (complete case analysis) and 274 (after multiple imputation) Multivariable robust Poisson regression models including maternal age, BMI, parity, chronic condition, gestational diabetes, preeclampsia, induction of labour, duration of labour, instrumental delivery, episiotomy. *Measures of effect modification*: on additive scale Relative Excess Risk due to Interaction RERI (95% CI) 0.1250 (0.0321–0.2179); on multiplicative scale Ratio of RRs (95% CI) 1.14 (0.95–1.36) Abbreviations: aRR, adjusted risk ratio; EPDS, Edinburgh Postnatal Depression Scale; PPD, post‐partum depression; RR, risk ratio.

^a^ Unadjusted risk ratio.

^b^ Complete case analysis.

^c^ After multiple imputation.

^d^ EPDS < 11.

^e^ 11 ≤ EPDS < 13.

^f^ EPDS ≥ 13.

^g^ 111 women with missing data for maternal place of birth.

Considering the severity of depression symptoms, 139 (5.2%) women had moderate depressive symptoms (11 ≤ EPDS < 13) and 230 (8.6%) severe depressive symptoms (EPDS ≥ 13). Post‐partum haemoglobin was associated with moderate depressive symptoms (adjusted RR 0.84; 95% CI, 0.72–0.97) but not with severe depressive symptoms (adjusted RR 0.95; 95% CI, 0.84–1.07) (Table 3).

Amongst the 2287 European‐born women, 934 (42.7%) had post‐partum anaemia and 282 (12.3%) had symptoms of PPD. In this group, we found similar patterns of association as in the main analysis.

Amongst the 274 non‐European‐born women, 133 (51.2%) had post‐partum anaemia and 63 (23.0%) had symptoms of PPD. In this group, haemoglobin was not associated with symptoms of PPD (adjusted RR 0.97; 95% CI, 0.77–1.24), regardless of PPD symptom severity level (Table 3).

Profile of maternal anaemia was different according to maternal place of birth: 25.6% of non‐European‐born women had gestational anaemia, versus 15.6% of European‐born women (Table S3).

In the first sensitivity analysis with inverse probability weighing to account for potential selection bias due to attrition, the weighted prevalence of PPD symptoms was 14.4% (13.1–15.9) and the results of the association analysis were similar to those of the main analysis (Table S4).

In the second sensitivity analysis, after exclusion of the 309 (11.6%) women with post‐partum haemorrhage, 869 (38.5%) women had post‐partum anaemia (Table S5) and 319 (13.5%) had symptoms of PPD (Table S6). Patterns of association between post‐partum haemoglobin and symptoms of PPD were similar to the main analysis (Table S6).

In the third sensitivity analysis, post‐partum haemoglobinaemia was associated with the EPDS score. After adjustment for potential confounding factors, the EPDS score decreased by 0.23 for each 1 g/dL increase in post‐partum haemoglobinaemia (Table S7).

## 5 Discussion

### 5.1 Main Findings

In this prospective study of women with vaginal birth, 43.6% had anaemia in the immediate post‐partum and 13.8% had symptoms of PPD two months after delivery. Immediate post‐partum haemoglobin was associated with PPD symptoms at two months. Each 1 g/dL increase in haemoglobin level was associated with a 9% decrease in the risk of post‐partum depression symptoms. When differentiating two levels of PPD severity, haemoglobin was specifically associated with moderate PPD symptoms, with a 16% decrease for each 1 g/dL increase in haemoglobin, but not with severe PPD symptoms. In European‐born women, similar associations were found. In contrast, in non‐European‐born women, the prevalence of PPD symptoms was much higher, and immediate post‐partum haemoglobin was not associated with PPD symptoms.

### 5.2 Strengths and Limitations

The strengths of our study include its prospective design, with clear temporality between the exposure and the outcome, the rigorous assessment for all women included of maternal haemoglobin at day 2 post‐partum and EPDS score 2 months after delivery, both collected systematically as planned in the TRAAP trial's protocol. Moreover, our sample size is one of the most important of the existing literature exploring this association: in a meta‐analysis including 8 studies on the association between post‐partum anaemia and PPD, 2574 women were included in all the studies combined [9], fewer than in our study. Additionally, we analysed maternal haemoglobin as a continuous variable, while previous studies considered anaemia as a binary variable. This enabled us to show its linear relationship with the risk of PPD symptoms, a novel finding in favour of a causal association. In a sensitivity analysis, we also considered the EPDS score as a continuous variable. We found a linear relationship between maternal haemoglobin and EPDS score. Finally, we considered the obstetrical context in the analysis: obstetrical complications and interventions can impact both maternal haemoglobin and post‐partum depressive symptoms, inducing a major confounding bias [29]. We used multiple approaches to limit this bias. First, our study only included women with vaginal delivery, so the mode of delivery could not have biassed our analyses. Second, the prospective collection of obstetrical data enabled us to adjust on potential confounding factors. Finally, we performed a sensitivity analysis after exclusion of the women with post‐partum haemorrhage, which reinforces the validity of our results.

Our study has some limitations. First, 28.8% of the women were not respondents to EPDS at 2 months post‐partum and were therefore excluded. However, the 71.2% response rate is still satisfactory compared to those previously reported in studies using self‐administered questionnaires to assess PPD [30, 31]. This attrition may have introduced selection bias since some pre‐existing and obstetric characteristics differ between non‐respondents and respondents. To mitigate this potential bias, we applied inverse probability weighting, which yielded unchanged results. This supports the robustness of our findings, suggesting they are unlikely to be influenced by selection bias.

Second, our source population was the women included in a randomised controlled trial. Socially vulnerable women were then probably underrepresented in our study population, although the main characteristics of the TRAAP trial population are similar to those of women with vaginal birth in France [32]. However, this does not limit the validity of our results on the association between maternal haemoglobin and PPD.

Third, our study assessed PPD symptoms with the EPDS self‐administered questionnaire, which provides a screening for PPD but not a definite diagnosis; this latter requiring a clinical evaluation by a qualified provider. However, the EPDS is a validated and widely used screening tool, specifically designed to assess depression symptoms in the perinatal period, both in research and clinical practice [22]. Additionally, the feasibility and cost of clinical interviews restrict their use in large studies.

Finally, we have no information on the prescription of iron supplementation and on haemoglobin levels at two months post‐partum, at the time of EPDS completion. Therefore, it is unclear whether women were still anaemic.

### 5.3 Interpretation

The association between maternal anaemia and maternal morbidity and mortality is documented in the existing literature [33, 34]. However, regarding maternal mental health, the association between maternal anaemia and PPD is inconsistent [17]. Some studies reported an association [35, 36], with odd ratios up to 4, while others found no association [37]. Definitions of anaemia and of PPD differed wildly between studies. Defining anaemia in pregnant women is challenging [38]. The threshold used to define anaemia varied from 10.5 to 12 g/dL, and the timing of haemoglobin assessment ranged from the beginning of pregnancy to several weeks after delivery. PPD diagnosis tool, cut‐off values and timing of assessment also differed between studies. The important heterogeneity in the definitions of both the exposure and the outcome may account for these discrepancies. Moreover, the obstetrical context was often not considered in the analyses, leading to a potential major confounding bias.

This study, which involved systematic prospective data collection and consistent definitions of anaemia and PPD symptoms, found an association between post‐partum haemoglobin and PPD symptoms in women giving birth vaginally. These results are consistent with studies conducted on non‐pregnant populations reporting an association between anaemia and depression symptoms, which reinforces our results [39, 40].

Several biological mechanisms support the association between anaemia and depression. Low haemoglobin leads to increased cerebral blood flow, as a compensatory neuroprotection mechanism to protect the brain from hypoxia. The cerebral blood flow is particularly increased in frontal, middle temporal, and hippocampal regions which are involved in depression pathways [41]. Moreover, anaemia may induce dyspnea and fatigue, which reduce social activity and may contribute to the onset of depressive symptoms [42]. Furthermore, iron is involved in the metabolism of dopamine, serotonin, and GABA which are implicated in depression pathogenesis [15]. Iron status could then influence emotional behaviours and mental health by altering neurotransmitter homeostasis.

In this study, post‐partum haemoglobin was associated with moderate PPD symptoms but not with severe PPD symptoms. We can hypothesise that in women with severe PPD symptoms, other risk factors such as adverse life experiences might have a greater impact on PPD risk and overshadow a possible effect of haemoglobin.

Contrary to European‐born women, in non‐European‐born women, haemoglobin was not associated with PPD symptoms. In our study, most non‐European‐born women were from Africa. In these women, other risk factors such as social vulnerability might have outweighed the role of haemoglobin. Another possible explanation is that in non‐European‐born women, anaemia is more frequently chronic [10]. Preconception was not available in this study. However, the higher prevalence of gestational anaemia in non‐European‐born women suggests a possible higher prevalence of chronic anaemia in these women. Chronic anaemia could be better tolerated than acute anaemia, with less impact on emotional wellbeing, as reported for somatic symptoms [43]. Finally, in non‐European‐born women, anaemia is more often caused by other factors than iron deficiency, such as haemoglobinopathy [44, 45]. Since iron is implicated in the pathophysiology of depression [15], anaemia without iron deficiency might have less impact on maternal mental health. In previous studies, maternal place of birth was not considered in the analysis.

Our results highlight the importance of precise characterisation of PPD risk factors. They suggest that some risk factors are specific to some population subgroups or some profiles of PPD symptoms. Additionally, this study suggests that modelling haemoglobin levels as a continuous variable, rather than categorising them as anaemia, may offer a more comprehensive characterisation of its impact on clinical outcomes.

Although the strength of association between haemoglobinemia and PPD symptoms is moderate, and acknowledging that the causal nature of the association cannot be ensured, the identification of post‐partum anaemia appears as an opportunity to intervene, to possibly mitigate the risk of PPD. Nonetheless, experimental studies are required to assess the impact of iron supplementation on PPD incidence, especially given the frequency of iron supplementation side effects that may compromise women's adherence to this treatment [46]. Our results emphasise the potential importance of post‐partum anaemia on maternal health and support the 2017 WHO Global Nutrition target of a 50% reduction of anaemia prevalence in women of reproductive age by 2025 [47]. Despite such a high prevalence, international guidelines on post‐partum anaemia are highly heterogenous [48]. This study highlights the need for more consistency and evidence‐based recommendations in terms of preventing, screening, treatment, and follow‐up of post‐partum anaemia.

## 6 Conclusion

In this prospective study of women with vaginal birth in a high‐income country, immediate post‐partum haemoglobin was associated with PPD symptoms at 2 months, with a 10% decrease in the risk of PPD symptoms for each 1 g/dL increase in haemoglobin. These results emphasise the importance of precise characterisation of PPD risk factors to improve its prevention. This study paves the way to future interventional studies assessing the impact of post‐partum anaemia correction on women's post‐partum mental health.

## Author Contributions

Concept and design: C.D.‐T., L.S. Acquisition, analysis and interpretation of the data: L.L., C.D.‐T., L.S., A.F., M.P.B., S.T. and A.S. Drafting of the manuscript: L.L., C.D.‐T. and L.S. Critical revision of the manuscript: A.F., S.T., M.P.B., A.S. Statistical analysis: L.L., C.D.‐T., A.S. Supervision: C.D.‐T., L.S.

## Ethics Statement

The TRAAP trial protocol was approved by the Ouest II Committee for the Protection of Research Subjects and the French Health Products Safety Agency; 17 November 2014; TRAAP ClinicalTrials.gov number, NCT02302456. No additional ethical approval was required for this analysis.

## Conflicts of Interest

L.S. performed consultancy work and was a lecturer for Ferring Laboratories, GlaxoSmithKline, and Bayer. Moreover, L.S. has been a lecturer for Norgine for 3 years. M.P.B. has received honoraria from Novonordisk and 3 M Health Care. The other authors report no conflicts of interest.

## Supporting information


**Figure S1.** Relation between immediate post‐partum haemoglobin and post‐partum depression symptoms at 2 months, Poisson regression with fractional polynomial modelling adjusted for covariables.
**Figure S2**. Directed Acyclic Graph of the relation between haemoglobin in the immediate post‐partum and PPD symptoms at 2 months.
**Figure S3**. Prevalence of post‐partum depression symptoms at 2 months post‐partum according to haemoglobin in the immediate post‐partum (*n* = 2672).
**Table S1**. Characteristics of women with complete data compared with women with missing data for any covariate of the multivariable model.
**Table S2**. Characteristics of women who did or did not respond to the EPDS two months after delivery.
**Table S3**. Post‐partum haemoglobinemia and anaemia prevalence according to place of birth.
**Table S4**. First sensitivity analysis—Association between haemoglobin in the immediate post‐partum and depression symptoms at 2 months post‐partum, overall and according to maternal place of birth, with inverse probability weighting (IPW) of respondents to correct for non‐response at 2 months.
**Table S5**. Second sensitivity analysis amongst women without post‐partum haemorrhage (*n* = 2363)– Haemoglobinemia in the immediate post‐partum.
**Table S6**. Second sensitivity analysis amongst women without postpartum haemorrhage (*n* = 2363). Association between haemoglobin level in the immediate post‐partum period and post‐partum depression symptoms at 2 months post‐partum.
**Table S7**. Third sensitivity analysis ‐Association between haemoglobin in the immediate post‐partum and EPDS score as a continuous variable at 2 months post‐partum (*n* = 2672).

**Appendix S1.** Members of the TRAAP study group.

## Data Availability

The data that support the findings of this study are available from the corresponding author upon reasonable request.
