# Supplementary material for: Association Between Post‐Partum Anaemia and Depressive Symptoms at Two Months After Vaginal Delivery: A Secondary Analysis of the TRAAP Trial
Source: BJOG. 2025 Jul 14;132(11):1644–54. doi: 10.1111/1471-0528.18289 (PMC12411663; doi:10.1111/1471-0528.18289)
Supplement: Supplementary file 1 — Figure S1. Relation between immediate post‐partum haemoglobin and post‐partum depression symptoms at 2 months, Poisson regression with fractional polynomial modelling adjusted for covariables. Figure S2. Directed Acyclic Graph of the relation between haemoglobin in the immediate post‐partum and PPD symptoms at 2 months. Figure S3. Prevalence of post‐partum depression symptoms at 2 months post‐partum according to haemoglobin in the immediate post‐partum (n = 2672). Table S1. Characteristics of women with complete data compared with women with missing data for any covariate of the multivariable model. Table S2. Characteristics of women who did or did not respond to the EPDS two months after delivery. Table S3. Post‐partum haemoglobinemia and anaemia prevalence according to place of birth. Table S4. First sensitivity analysis—Association between haemoglobin in the immediate post‐partum and depression symptoms at 2 months post‐partum, overall and according to maternal place of birth, with inverse probability weighting (IPW) of respondents to correct for non‐response at 2 months. Table S5. Second sensitivity analysis amongst women without post‐partum haemorrhage (n = 2363)– Haemoglobinemia in the immediate post‐partum. Table S6. Second sensitivity analysis amongst women without postpartum haemorrhage (n = 2363). Association between haemoglobin level in the immediate post‐partum period and post‐partum depression symptoms at 2 months post‐partum. Table S7. Third sensitivity analysis ‐Association between haemoglobin in the immediate post‐partum and EPDS score as a continuous variable at 2 months post‐partum (n = 2672). [file BJO-132-1644-s001.docx]

Figure S1: Relation between immediate postpartum hemoglobin and postpartum depression symptoms at 2 months, Poisson regression with fractional polynomial modeling adjusted for covariables


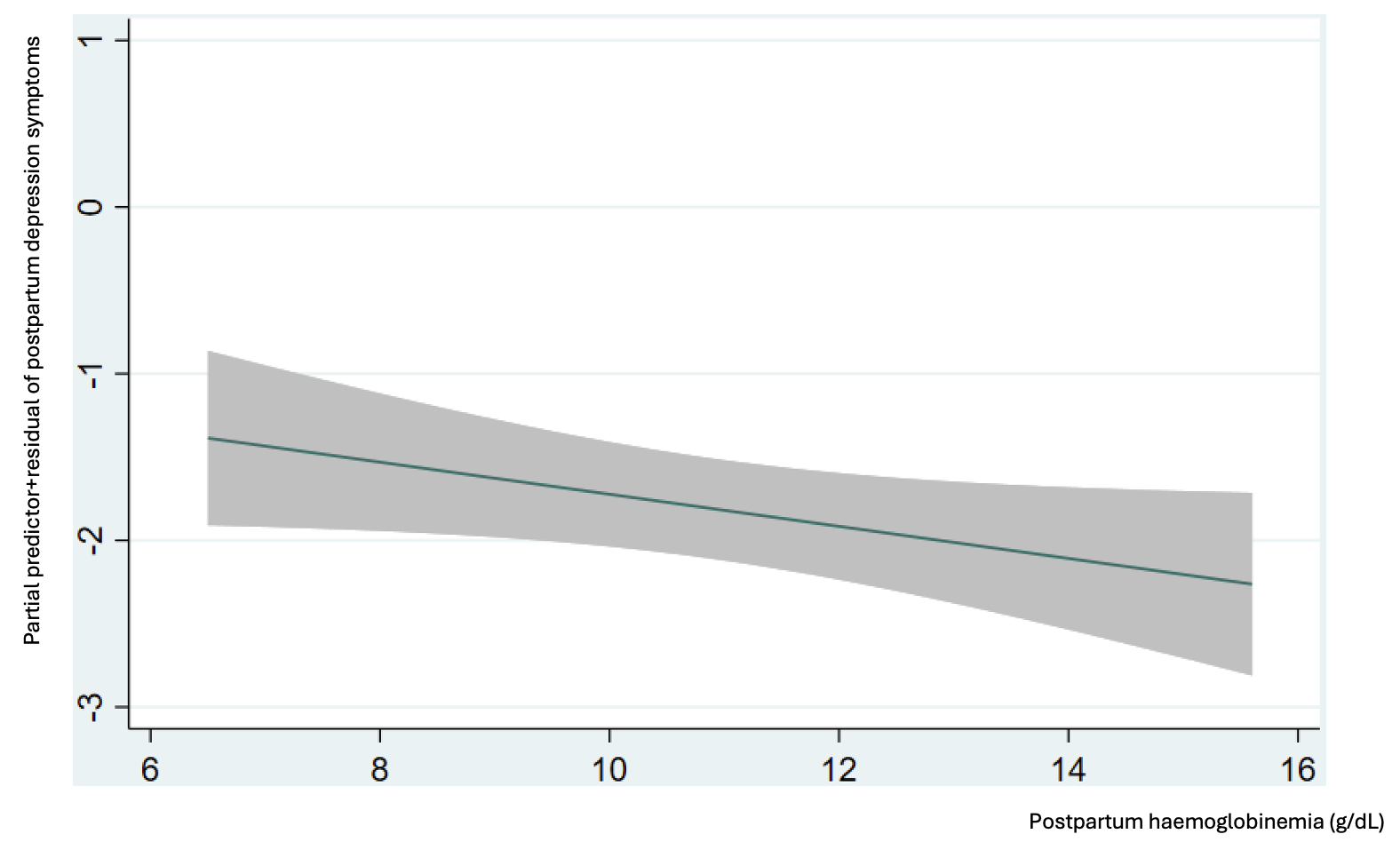


Covariables: maternal age, body mass index, parity, place of birth, chronic condition, gestational diabetes, preeclampsia, induction of labor, duration of labor, instrumental delivery and episiotomy.

Postpartum depression symptoms: EPDS ≥ 11 at 2 months postpartum

Postpartum haemoglobinemia: Latest maternal haemoglobin measured within 5 days following delivery, in 6 categories

N=2195

Figure S2: Directed Acyclic Graph of the relation between haemoglobin in the immediate postpartum and PPD symptoms at 2 months


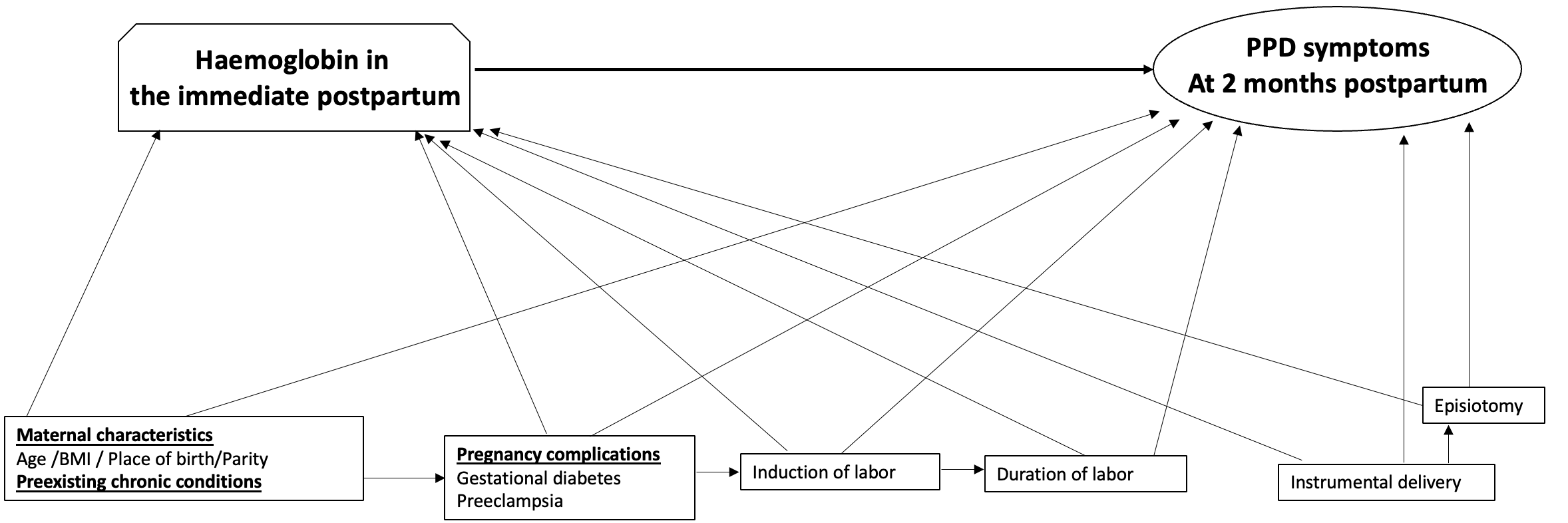


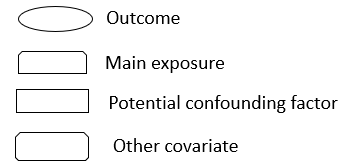


Figure S3: Prevalence of postpartum depression symptoms at 2 months postpartum according to haemoglobin in the immediate postpartum (n=2,672)


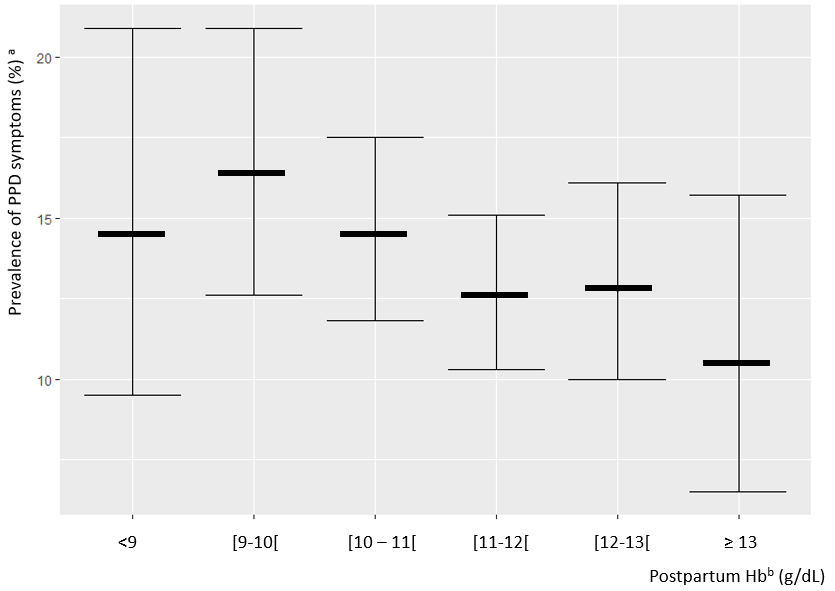


^a^ EPDS ≥ 11 at 2 months postpartum

^b^ Latest maternal haemoglobin measured within 5 days following delivery, in 6 categories

Table S1: Characteristics of women with women with complete data compared with women with missing data for any covariate of the multivariable model

|  | | Women with complete  data  N=2195 | | | Women with missing data for any covariate of the multivariable model  n=477 | | |
| --- | --- | --- | --- | --- | --- | --- | --- |
| Maternal characteristics |  | |  |  | |  |  |
| Age >=35 years | | 298 (14.3) | | | 75 (16.7) | | |
| BMI>30 kg.m^-2^ | | 49 (2.2) | | | 10 (2.2) | | |
| Place of birth | |  | | |  | | |
| Europe | | 1950 (88.8) | | | 337 (92.1) | | |
| Other | | 245 (11.2) | | | 29 (7.9) | | |
| Preexisting conditions | |  | | |  | | |
| Chronic hypertension | | 10 (0.5) | | | 3 (0.6) | | |
| Preexisting diabetes | | 11 (0.5) | | | 1 (0.2) | | |
| Obstetrical history | |  | | |  | | |
| Previous abortion | | 271 (12.4) | | | 65 (13.6) | | |
| Parity | |  | | |  | | |
| Nulliparous | | 1219 (55.5) | | | 266 (55.8) | | |
| Multiparous without previous cesarean | | 853 (38.9) | | | 184 (38.6) | | |
| Multiparous with previous cesarean | | 123 (5.6) | | | 27 (5.7) | | |
| Pregnancy complications | |  | | |  | | |
| Thrombopenia | | 100 (4.5) | | | 18 (3.8) | | |
| Gestational diabetes | | 240 (10.9) | | | 40 (8.4) | | |
| Preeclampsia | | 13 (0.6) | | | 4 (0.8) | | |
| Labor, delivery and neonatal characteristics |  | |  |  | |  |  |
| Instrumental vaginal delivery | | 405 (18.5) | | | 80 (16.8) | | |
| Blood loss ≥ 500 milliliters^a^ | | 226 (10.3) | | | 33 (6.9) | | |
| Birthweight ≥ 4000 grams | | 170 (7.7) | | | 33 (6.9) | | |
| 5 minutes APGAR<7 | | 16 (0.7) | | | 3 (0.6) | | |
| Neonatal resuscitation | | 73 (3.3) | | | 10 (2.1) | | |
| Neonatal death | | 0 (0) | | | 0 (0) | | |
| Postpartum period |  | |  |  | |  |  |
| Postpartum Hb (g/dL) | |  | | |  | | |
| >=11 | | 1230 (56.0) | | | 215 (58.9) | | |
| [10-11[ | | 526 (24.0) | | | 95 (26.0) | | |
| <10 | | 439 (20.0) | | | 55 (15.1) | | |
| Bad memories of childbirth at day 2^b^ | | 51(2.62) | | | 16 (4.5) | | |
| Rehospitalization in the 12 weeks following delivery | | 23 (1.1) | | | 2 (0.4) | | |

^a^ Quantified with a graduated collector bag ^b^ Experience of childbirth was assessed with a self-administered questionnaire on day 2 after delivery with the question “Today, what are your memories of childbirth?” Women answered this question with a five-point Likert-type scale (excellent, good, intermediate, bad, or very bad). Bad memories of childbirth were defined by a response of “bad” or “very bad”.

Table S2: Characteristics of women who did or did not respond to the EPDS two months after delivery

|  | | Women respondent to EPDS  n=2,672 | | | Women non-respondent to EPDS  n=1,082 | | |
| --- | --- | --- | --- | --- | --- | --- | --- |
| Maternal characteristics |  | |  |  | |  |  |
| Age >=35 years (n=3,754) | | 506 (18.9) | | | 188 (17.4) | | |
| BMI>30 kg.m^-2^ (n=3,720) | | 219 (8.5) | | | 115 (10.8) | | |
| Place of birth (n=3,558) | |  | | |  | | |
| Europe | | 2,287 (89.3) | | | 662 (66.4) | | |
| Other | | 274 (10.7) | | | 335 (33.6) | | |
| Preexisting conditions (n=3,754) | |  | | |  | | |
| Any preexisting condition | | 27 (1.0) | | | 12 (1.1) | | |
| Chronic hypertension | | 13 (0.5) | | | 8 (0.7) | | |
| Diabetes | | 12 (0.5) | | | 4 (0.4) | | |
| Obstetrical history | |  | | |  | | |
| Previous abortion (n=3,754) | | 336 (12.6) | | | 213 (19.7) | | |
| Parity (n=3,754) | |  | | |  | | |
| Nulliparous | | 1,485 (55.6) | | | 513 (47.4) | | |
| Multiparous without previous cesarean | | 1,037 (38.8) | | | 493 (45.6) | | |
| Multiparous with previous cesarean | | 150 (5.6) | | | 76 (7.0) | | |
| Pregnancy complications (n=3,754) | |  | | |  | | |
| Thrombopenia | | 118 (4.4) | | | 52 (4.8) | | |
| Gestational diabetes | | 280 (10.5) | | | 123 (11.4) | | |
| Preeclampsia | | 17 (0.6) | | | 10 (0.9) | | |
| Labor, delivery and neonatal characteristics |  | |  |  | |  |  |
| Instrumental vaginal delivery (n=3,754) | | 485 (18.2) | | | 173 (16.0) | | |
| Blood loss ≥ 500 milliliters^a^ (n=3,754) | | 259 (9.7) | | | 88 (8.1) | | |
| Birthweight ≥ 4000 grams (n=3,754) | | 203 (7.6) | | | 95 (8.8) | | |
| 5 minutes APGAR<5 (n=3,747) | | 19 (0.7) | | | 9 (0.8) | | |
| Neonatal resuscitation (n=3,754) | | 83 (3.1) | | | 42 (3.9) | | |
| Neonatal death (n=3,754) | | 0 (0) | | | 1 (0.1) | | |
| Postpartum period |  | |  |  | |  |  |
| Postpartum Hb (n=3,578) (g/dL) | |  | | |  | | |
| >=11 | | 1,445 (56.5) | | | 501 (49.2) | | |
| [10-11[ | | 621 (24.3) | | | 255 (25.1) | | |
| <10 | | 494 (19.3) | | | 262 (25.7) | | |
| Bad memories of childbirth at day 2^b^ (n=3,073) | | 67 (2.9) | | | 20 (2.6) | | |
| Rehospitalization in the 12 weeks following delivery (n=3,560) | | 25 (1.0) | | | 9 (1.0) | | |

^a^ Quantified with a graduated collector bag ^b^ Experience of childbirth was assessed with a self-administered questionnaire on day 2 after delivery with the question “Today, what are your memories of childbirth?” Women answered this question with a five-point Likert-type scale (excellent, good, intermediate, bad, or very bad). Bad memories of childbirth were defined by a response of “bad” or “very bad”.

BMI : body mass index, dL : deciliter, g : grams, Hb : haemoglobin

Table S3: Postpartum haemoglobinemia and anaemia prevalence according to place of birth

|  | **European-born women**  n=2,287 | **Non-European-born women**  n=274 | | | |
| --- | --- | --- | --- | --- | --- |
|  | |  |  | |  |
| **Maternal anaemia** (Hb<11g/dL) n=2,364 |  |  | |  |  |
| None | 1,121 (52.9) | 105 (42.7) | | | |
| Gestational^a^ without postpartum anaemia | 94 (4.4) | 15 (6.1) | | | |
| Postpartum^b^ without gestational anaemia | 66 (31.4) | 78 (31.7) | | | |
| Gestational and postpartum anaemia | 237 (11.2) | 48 (19.5) | | | |
| **Postpartum haemoglobin**^b^ (g/dL) median (IQR) n=2,450 | 11.2 (10.3 – 12) | 10.9 (10 – 11.9) | | | |
| **Postpartum anaemia** n=2,450 (Hb<11g/dL) | 934 (42.7) | 133 (51.2) | |  |  |
| **Postpartum haemoglobin** (g/dL) n=2,450 |  |  | | | |
| ≥11 | 1,256 (57.4) | 127 (48.9) | | | |
| [10-11[ | 520 (23.7) | 71 (27.3) | | | |
| <10 | 414 (18.9) | 62 (23.9) | | | |
|  |  |  | | | |

^a^ Haemoglobinemia routinely measured as part of the prenatal care in the 8th month of pregnancy of less than 11 g/dL. If haemoglobinemia of the 8th month was unavailable, we considered the haemoglobinemia measured in the 6th month. Available for 2,253 women.

^b^ Defined as the latest maternal haemoglobin measured in the 5 days following delivery. In case of red blood cells transfusion, haemoglobin after transfusion was considered. Among the 2,561 women included in the analysis, 304 had missing data on postpartum haemoglobin.

dL: deciliter, g: grams, IQR: interquartile range

Table S4: First sensitivity analysis - Association between haemoglobin in the immediate postpartum and depression symptoms at 2 months postpartum, overall and according to maternal place of birth, with inverse probability weighting (IPW) of respondents to correct for non-response at 2 months

|  | Corrected prevalence of  the outcome  % (95% CI) | RR*  *For each 1g/dL*  *increase in Hb* | aRR†  *For each 1g/dL*  *increase in Hb* |
| --- | --- | --- | --- |
| **Overall population** n=2672 |  |  |  |
| **PPD symptoms** (EPDS ≥11) | 14.4 (13.1 – 15.9) | 0.93 [0.86-0.997] | 0.90 [0.82-0.99] |
| **PPD symptoms** |  |  |  |
| None§ | 85.6 (84.1-86.9) | Ref | Ref |
| Moderate symptoms¶ | 5.4 (4.6-6.4) | 0.90 [0.78-1.03] | 0.80 [0.69-0.94] |
| Severe symptoms** | 9.0 (7.9-10.2) | 0.93 [0.84-1.02] | 0.94 [0.82-1.08] |
| **According to maternal place of birth**†† | |  |  |
| **European-born women n=2287** | | |  |
| **PPD symptoms** (EPDS ≥11) | 12.4 (11.1-13.8) | 0.92 [0.85-1.00] | 0.90 [0.82-0.98] |
| **PPD symptoms** |  |  |  |
| None§ | 87.6 (86.2 – 88.9) | Ref | Ref |
| Moderate symptoms¶ | 4.9 (4.1-5.9) | 0.86 [0.75-0.99] | 0.78 [0.67-0.92] |
| Severe symptoms** | 7.5 (6.4-8.6) | 0.95 [0.84-1.07] | 0.96 [0.84-1.09] |
| **Non-european-born women n=274** | | |  |
| **PPD symptoms** (EPDS ≥11) | 23.3 (18.4-29.0) | 1.03 [0.87-1.21] | 0.98 [0.81-1.18] |
| **PPD symptoms** |  |  |  |
| None§ | 76.7 (71.0-81.6) | Ref | Ref |
| Moderate symptoms¶ | 7.6 (4.8-11.7) | 1.19 [0.78-1.80] | 1.15 [0.70-1.90] |
| Severe symptoms** | 15.7 (11.7-20.8) | 0.96 [0.76-1.22] | 0.87 [0.65-1.16] |

aRR: adjusted risk ratio, EPDS: Edinburgh Postnatal Depression Scale, PPD: Postpartum Depression, RR: risk ratio
* Unadjusted risk ratio  † Complete case analysis
§ EPDS <11 ¶11≤EPDS<13 ** EPDS ≥ 13

**Overall population** n=2195
multivariable robust Poisson regression model including maternal age, BMI, parity, place of birth, chronic condition, gestational diabetes, preeclampsia, induction of labor, duration of labor, instrumental delivery, episiotomy.
**Analysis according to maternal place of birth**multivariable robust Poisson regression model including maternal age, BMI, parity, chronic condition, gestational diabetes, preeclampsia, induction of labor, duration of labor, instrumental delivery, episiotomy.  **European-born women** n=1950 **Non-European-born women** n=243
†† 111 women with missing data for maternal place of birth

Table S5: Second sensitivity analysis among women without postpartum haemorrhage (n=2,363)

– Haemoglobinemia in the immediate postpartum

| **Haemoglobinemia in the immediate postpartum**^a^ | **n (%)** |
| --- | --- |
| **Postpartum haemoglobin** (g/dL) median (IQR) | 11.3 (10.5 – 12.1) |
| **Postpartum anaemia** (Hb<11g/dL) | 869 (38.5) |
| **Postpartum haemoglobin** (g/dL) |  |
| ≥11 | 1388 (61.5) |
| [10-11[ | 552 (24.5) |
| <10 | 317 (14.1) |

^a^ Defined as the latest maternal haemoglobin measured in the 5 days following delivery. In case of red blood cells transfusion (n=20), haemoglobin after transfusion was taken into account. Among the 2,363 women included in this analysis, 106 women (4.5%) had missing data on postpartum haemoglobin

g: grams, dL: deciliter, IQR: interquartile range

Table S6: Second sensitivity analysis among women without postpartum haemorrhage (n=2,363)– Association between haemoglobin level in the immediate postpartum period and postpartum depression symptoms at 2 months postpartum

|  | Prevalence of  the outcome  n (%) | RR^a^  *For each 1g/dL*  *increase in Hb* | aRR^b^  *For each 1g/dL*  *increase in Hb* | aRR^c^  *For each 1g/dL*  *increase in Hb* |
| --- | --- | --- | --- | --- |
|  |  |  |  |  |
| **Overall population** |  |  |  |  |
| **PPD symptoms** (EPDS ≥11) | 319 (13.5) | 0.91 (0.83 – 1.01) | 0.89 (0.78 – 1.01) | 0.90 (0.82 – 1.00) |
| **PPD symptoms** |  |  |  |  |
| None^d^ | 2044 (86.5) | Ref | Ref | Ref |
| Moderate symptoms^e^ | 118 (5.0) | 0.88 (0.76 – 1.03) | 0.80 (0.65 – 0.97) | 0.83 (0.69-0.99) |
| Severe symptoms^f^ | 201 (8.5) | 0.93 (0.82 – 1.05) | 0.95 (0.81 – 1.11) | 0.94 (0.81 – 2.20) |
|  | |  |  |  |
|  | |  |  |  |

aRR: adjusted risk ratio, EPDS: Edinburgh Postnatal Depression Scale, PPD: Postpartum Depression, RR: risk ratio
Multivariable Poisson regression model including maternal age, BMI, parity, place of birth, chronic condition, gestational diabetes, preeclampsia, induction of labor, duration of labor, instrumental delivery, episiotomy.
^a^ Unadjusted relative risk 
^b^ Complete case analysis n=1,931
^c^ After multiple imputation n=2,363

Table S7: Third sensitivity analysis -Association between haemoglobin in the immediate postpartum and EPDS score as a continuous variable at 2 months postpartum (n=2672)

| **Outcome** | **Median (IQR)** | **OR ***  For each 1g/dL  increase in Hb | **aOR**†  For each 1g/dL  increase in Hb | **aOR**‡  For each 1g/dL  increase in Hb |
| --- | --- | --- | --- | --- |
|  |  |  |  |  |
| **Continuous  EPDS score** | 5 (2-8) | -0.22 [-0.35 – -0.08] | -0.23 [-0.38 – -0.08] | -0.22 [-0.37 – -0.7] |

aOR: adjusted odd ratio, EPDS: Edinburgh Postnatal Depression Scale * ^*^Unadjusted odd ratio

† Complete case analysis n=2560 ‡ After multiple imputation n=2672
multivariable linear regression model including maternal age, BMI, parity, place of birth, chronic condition, gestational diabetes, preeclampsia, induction of labor, duration of labor, instrumental delivery, episiotomy.
