# Supplementary material for: Association Between Post‐Partum Anaemia and Depressive Symptoms at Two Months After Vaginal Delivery: A Secondary Analysis of the TRAAP Trial
Source: BJOG. 2025 Jul 14;132(11):1644–54. doi: 10.1111/1471-0528.18289 (PMC12411663; doi:10.1111/1471-0528.18289)
Supplement: Supplementary file 2 — Appendix S1. Members of the TRAAP study group. [file BJO-132-1644-s002.docx]

Appendix

**The TRAAP study group**

Loïc Sentilhes MD, PhD^1^, Norbert Winer MD, PhD^2,3^, Elie Azria MD, PhD^4, 5^, Marie Victoire Sénat MD, PhD^6^, Camille Le Ray MD, PhD^5,7^, Delphine Vardon MD^8^, Franck Perrotin MD, PhD^9^, Raoul Desbrière MD^10^, Florent Fuchs MD, PhD^11,12^, Gilles Kayem MD, PhD^5,13^, Guillaume Ducarme MD, PhD^14^, Muriel Doret-Dion MD, PhD^15^, Cyril Huissoud MD, PhD^16^, Caroline Bohec MD^17^, Philippe Deruelle MD, PhD^18^, Astrid Darsonval PharmD^19,20^, Jean-Marie Chrétien MSc^21^, Aurélien Seco MSc^5^, Valérie Daniel PharmD^19,20^, Catherine Deneux-Tharaux MD, PhD^5^.

1. Department of Obstetrics and Gynecology, Bordeaux University Hospital, Bordeaux, France.

2. Department of Obstetrics and Gynecology, University Medical Centre of Nantes; Centre d'Investigation Clinique CIC Mere enfant, University Hospital, Nantes, France.

3. National Institute of Agricultural Research (INRA), UMR 1280, Physiology of Nutritional Adaptations, University of Nantes, IMAD and CRNH-Ouest, Nantes 44000, France

4. Maternity unit, Paris Saint Joseph Hospital, Paris Descartes University, Paris, France.

5. Université Paris Cité, Centre for Research in Epidemiology and Statistics (CRESS) U1153, Obstetrical Perinatal and Paediatric Epidemiology Research Team (EPOPé), INSERM, INRAE, Paris, France

6. Department of Obstetrics and Gynecology, Bicetre University Hospital, Assistance Publique-Hôpitaux de Paris, Paris, France.

7. Port Royal Maternity Unit, Cochin Hospital, Assistance Publique-Hôpitaux de Paris, Paris, France; DHU Risks in Pregnancy, Paris, France; Paris Descartes University, Paris, France

8. Department of Obstetrics and Gynecology, Caen University Hospital, Caen, France

9. Department of Obstetrics and Gynecology, Tours University Hospital, Tours, France

10. Department of Obstetrics and Gynecology, Saint-Joseph Hospital, Marseille, France.

11. Department of Obstetrics and Gynecology, Montpellier University Hospital, France.

12. Inserm, CESP Centre for Research in Epidemiology and Population Health, U1018, Reproduction and child development, Villejuif, France.

13. Department of Obstetrics and Gynecology, Trousseau Hospital, Assistance Publique-Hôpitaux de Paris, Paris, France.

14. Department of Obstetrics and Gynecology, Centre Hospitalier Departemental, La Roche sur Yon, France.

15. Hospices Civils de Lyon, Hospital Femme-Mère-Enfant, Department of Obstetrics and Gynecology, University Lyon 1, France.

16. Department of Obstetrics and Gynecology, Croix Rousse University Hospital, Lyon, F-69004, France.

17. Department of Obstetrics and Gynecology, François Mitterrand Hospital, Pau, France.

18. Department of Obstetrics and Gynecology, Jeanne de Flandre University Hospital, Lille, France

19. Department of Pharmacy, Angers University Hospital, Angers, France.

20. PPRIGO (Production Pharmaceutique pour la Recherche Institutionnelle du Grand Ouest), Brest University Hospital, Brest, France.

21. Angers University Hospital, Department of Clinical Research, Angers University Hospital Angers, France.
